# Dual-attention-based recurrent neural network for hand-foot-mouth disease prediction in Korea

**DOI:** 10.1038/s41598-023-43881-6

**Published:** 2023-10-03

**Authors:** Sieun Lee, Sangil Kim

**Affiliations:** Department of Mathematics, Pusan National University, Busan, 46241 Republic of Korea

**Keywords:** Health policy, Health services

## Abstract

Hand–foot–mouth disease (HFMD) is a viral disease that occurs primarily in children. Meteorological factors have a significant impact on its popularity annually in Korea. This study proposes a new HFMD prediction model using a dual-attention-based recurrent neural network (DA-RNN) and important weather factors for HFMD in Korea. First, suspected cases of HFMD in each state were predicted using meteorological factors from the DA-RNN. Second, the weather factors were divided into six categories: temperature, wind, rainfall, day length, humidity, and air pollution to conduct sensitivity analysis. Because of this prediction, the proposed model showed the best performance in predicting the number of suspected HFMD cases in a week compared with other RNN methods. Sensitivity analysis showed that air pollution and rainfall play an important role in HFMD in Korea. This model provides information for HFMD prevention and control and can be extended to predict other infectious diseases.

## Introduction

Hand–foot–mouth disease (HFMD) is a viral disease caused by the transmission of coxsackievirus A16 (CoxA16) and enterovirus 71 (EV 71) into the intestine^1^. It mostly affects preschoolers and is caused by respiratory secretions and excretions from gathering places for children. Most of these diseases have mild symptoms; however, death occurs in severe cases^2,3^. Asia shows a recurring trend annually^4,5^. Because no specific treatment or vaccine is available, only treatments to alleviate symptoms in cases of infection have been performed^6^. Various causative organisms can re-infect the disease. Models capable of predicting disease patterns are essential for disease prevention.

Asia has periodic fluctuations that show a repetitive epidemic pattern, with the peak of the epidemic occurring every summer before COVID-19^7^. It is classified as a national infectious disease (Level 4) in Korea and is reported by the National Institute of Health through the sampling surveillance system. Suspected cases among 1000 patients are reported to the Korea Centers for Disease Control and Prevention on a weekly basis^8^. Previous research has demonstrated a significant correlation between HFMD and meteorological conditions such as average temperature, relative humidity, wind speed, and sunshine hours^9–11^. Among these meteorological conditions, it is important to determine the meteorological factors that should be prioritized.

Numerous studies have been conducted to generate HFMD predictive models^11–15^. A mathematical model was used to determine the seasonality of HFMD infectivity and to predict the number of patients, which showed that peaks occur annually in summer and autumn^13^. Long short-term memory (LSTM), autoregressive integrated moving average (ARIMA), and nonlinear autoregressive (NAR) neural networks have been used to determine the most appropriate model using machine learning and statistical methods. These methodologies accounted for the seasonal and trending characteristics of HFMD. However, these studies did not identify the factors that significantly influence seasonality^14^. Similarly, the time-series pattern was learned using the LSTM method to predict HFMD generation and determine the contribution of meteorological factors to spatiotemporal effects using a statistical method called geodetector^12^. In this study, we identified HFMD predictors and influential meteorological factors. However, the performance of the LSTM deteriorates as the length of the time series increases. Recently, models that predict epidemics in the form of extended LSTM using attention techniques have been developed^16,17^.

Numerous studies on forecasting models using meteorological factors have been conducted in China. In Korea, the epidemiological characteristics and spatiotemporal distribution of HFMD in children under six years of age were evaluated using national health insurance data, and the prevalence pattern was evaluated using the spatiotemporal clustering method^15^. However, meteorological factors that significantly affect the prevalence of HFMD in Korea have not yet been studied.

The limitations of previous studies were overcome by predicting HFMD using a dual attention-based recurrent neural network (DA-RNN) model^18^ with 20 meteorological factors. Through the training of this model, we identified important meteorological factors associated with HFMD in Korea that are unknown to our knowledge. This study provides the information necessary to establish national management and prevention strategies in the future. This model could be extended to predict other infectious diseases.

## Results

### Epidemiological and meteorological characteristics

**Figure 1:**
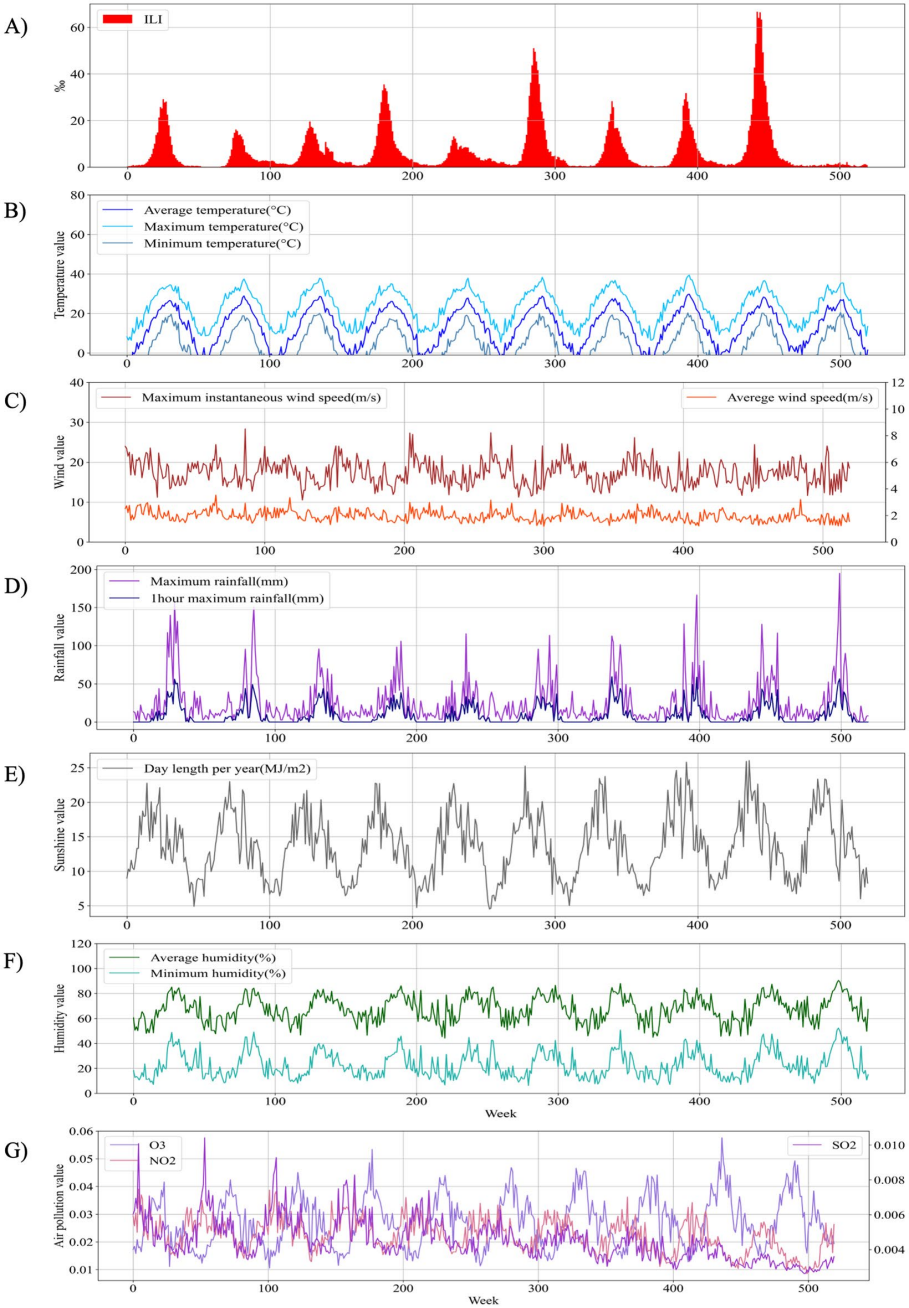
Epidemiological and meteorological characteristics. (**A**) Number of suspected HFMD cases per thousand people per week. (**B**) Average, maximum, and minimum temperatures among the temperature group. (**C**) Maximum instantaneous wind speed and average wind speed among the wind group. (**D**) Maximum rainfall and 1 h maximum rainfall among the rainfall group. (**E**) Day length per year among the sunshine group. (**F**) Average and minimum humidity among the humidity group. (**G**) SO_2_, O_3_, and NO_2_ among the air pollution group.

In Korea, HFMD was reported to exhibit an annual repeating pattern from 2011 to 2020. The most prevalent weeks were typically those between weeks 25 and 30. The other two are represented by 2015, which had week 22 as the popular, and 2020, which had week 38 as the popular week. The size of the prevalence varied significantly from year to year, with 2019 showing the highest level (66.7 thousand parts, 27 weeks) and 2020 showing the lowest level (2.2 thousand parts, 38 weeks). The HFMD statistics for each year are shown in Fig. 1A.

Table 2 shows 20 meteorological factors from six groups used from 2011 to 2020. Figure 1B–G show some meteorological factors by group. Figure 1 shows the epidemiological and meteorological characteristics from 2011 to 2020, and similar patterns can be identified for each period. Supplementary Table S1 shows the Pearson’s correlation analysis between epidemiological and meteorological characteristics.

### Estimation of suspected HFMD patients

**Figure 2:**
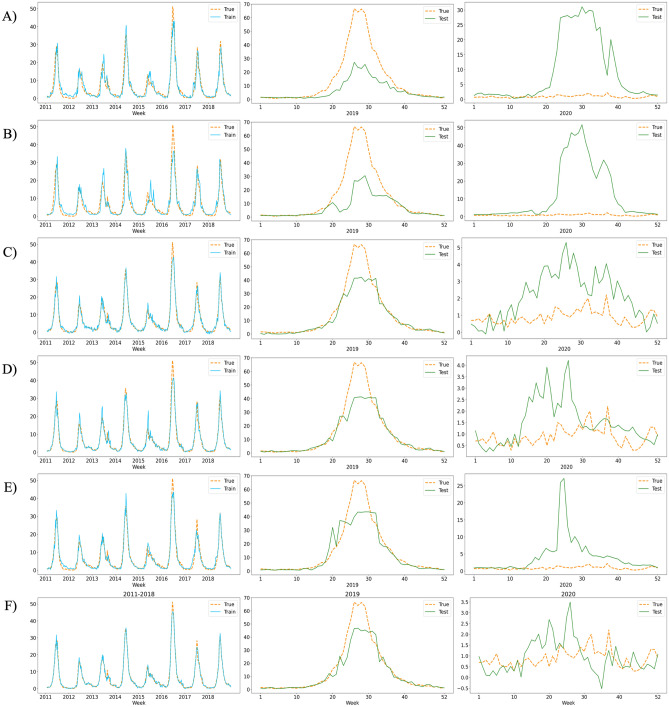
Estimation of suspected HFMD patients. In (**A**) LSTM; (**B**) GRU; (**C**) Seq2seq; (**D**) encoder attention-based seq2seq; (**E**) decoder attention-based seq2seq; (**F**) DA-RNN. The orange, blue, and green lines represent the actual data value, predicted value of the train, and predicted value of the test, respectively.

Twenty meteorological factors were normalized and used. Meteorological factors and weekly HFMD data from 2011 to 2018 were used as training sets for the DA-RNN. Meteorological factors and HFMD data from 2019 to 2020 were used as a test set to check the forecasting model, which was saved after 1000 epochs. The mean absolute error (MAE), root mean square error (RMSE), mean absolute percentage error (MAPE), and R-squared score (R^2^) were used to evaluate the performance of the experiment. Table 1 compares the performances of each method. DA-RNN achieved the best performance for all evaluation indicators. Figure 2 shows the training and test prediction results for LSTM, GRU, seq2seq, encoder attention-based seq2seq, decoder attention-based seq2seq, and DA-RNN.

**Table 1:** Prediction results performances.

Models	MAE	RMSE	MAPE	R^2^
LSTM	3.0114	6.8165	1.5292	0.5785
GRU	3.4922	8.2496	1.8420	0.3827
Seq2seq	1.5339	3.2754	0.6816	0.9026
Encoder attention	1.4555	3.2731	0.4587	0.8905
Decoder attention	1.7313	3.6694	0.9307	0.8778
DA-RNN	0.8544	2.7117	0.3163	0.9333

### Sensitivity analysis

**Figure 3:**
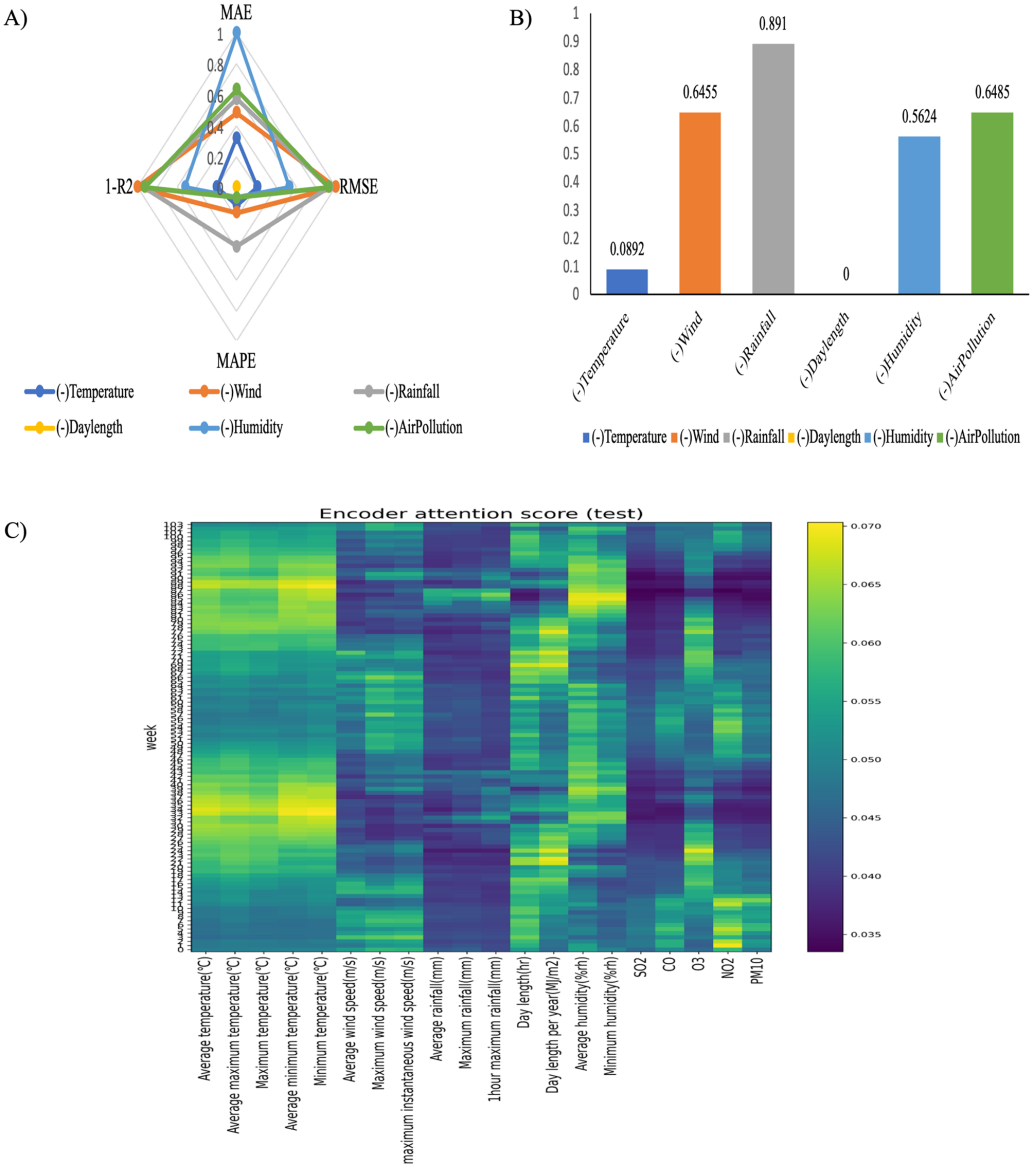
Sensitivity analysis results. (**A**) Spider map of the sensitivity analysis important value results; (**B**) Important scores; (**C**) Encoder attention scores of the DA-RNN test results.

The sensitivity test results are shown in Fig. 3. The sensitivity analysis compared the evaluation scores (MAE, RMSE, MAPE, and R^2^) by eliminating them by group, as shown in Table S2.

In order to determine important meteorological groups by objectifying the evaluation scores obtained from the sensitivity experiment, a spider map was obtained through the defined important values, which is shown in Fig. 3A. The importance score was calculated based on the area of this spider map, and the larger the value, the greater the importance, which is shown in Fig. 3B.

The predicted important score after removing the five elements from the temperature group was 0.0892. The predicted important score two elements of the wind group removed was 0.6455; the predicted important score with two elements of the rainfall group removed was 0.891; the predicted important score with two elements of the day length group deleted was 0; the important score with two elements of the humidity group omitted was 0.5624; and the important score with five elements of the air pollution group deleted was 0.6485. The important score results for each group are shown in Fig. 3B. Supplementary Fig. S2 shows the sensitivity analysis important score results.

Because of the characteristics of its structure, DA-RNN calculates the attention scores of features every time, which can confirm changes in the importance of meteorological factors. Figure 3C shows changes in the importance of meteorological factors during the test period. Although the score values vary depending on the period, rainfall and air pollution groups have high important scores over the overall time.

## Method

**Figure 4:**
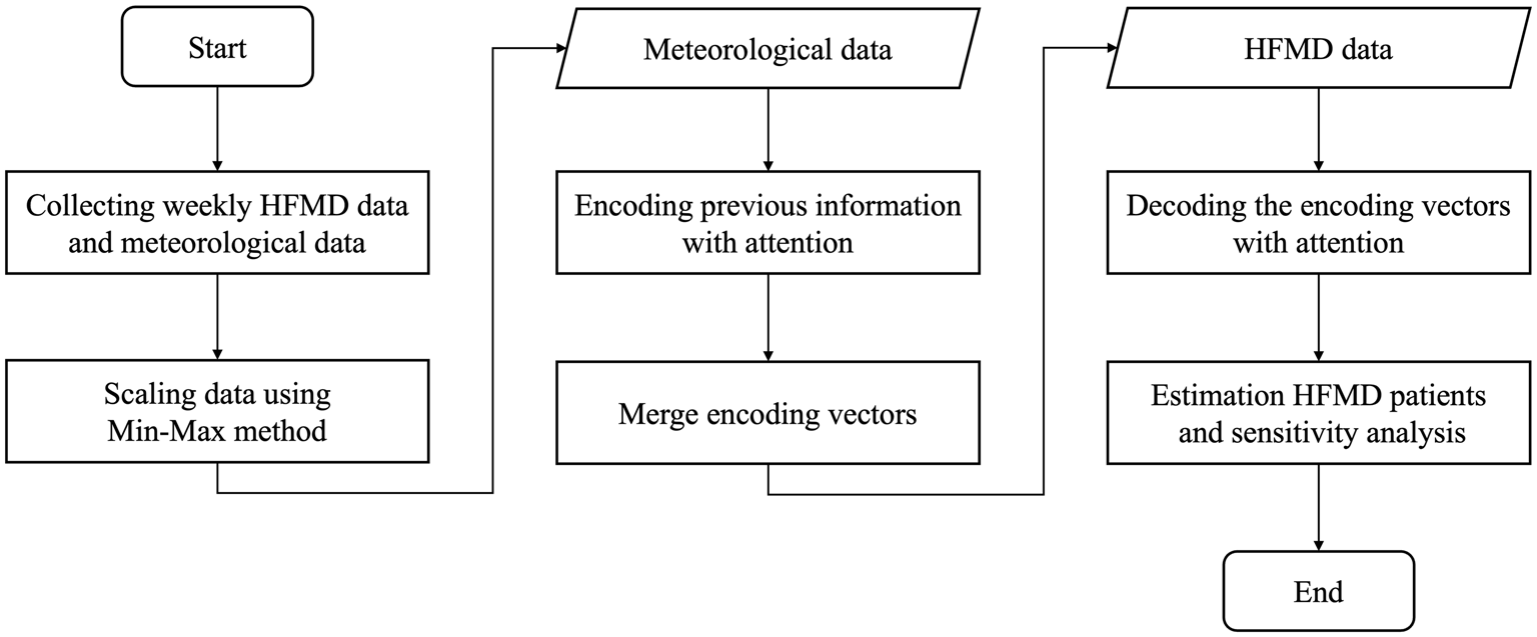
Framework diagram.

Figure 4 summarizes the HFMD prediction using DA-RNN. After data collection and normalization, weekly HFMD data were regression-targeted using DA-RNN to predict weekly suspected HFMD cases per 1000 people and to discriminate between important meteorological factor groups.

### Data collection

HFMD data were obtained from 2011 to 2020 from the number of suspected cases per 1,000 people per state provided by the Korea Centers for Disease Control and Prevention^19^. For meteorological factors, 15 types of data on temperature, wind speed, precipitation, day length, and humidity from 2011 to 2020 were collected using the Korea Meteorological Data Open Portal^20^. Five types of air pollution data were collected from 2011 to 2020 through AirKorea^21^.

Table 2 shows 20 meteorological factors and descriptions used for predicting HFMD. These meteorological factors were classified into six categories: (1) Temperature: average temperature (°C), average maximum temperature (°C), maximum temperature (°C), average minimum temperature (°C), and minimum temperature (°C); (2) Wind: average wind speed (m/s), maximum wind speed(m/s), and maximum instantaneous wind speed(m/s); (3) Rainfall: average rainfall (mm), maximum rainfall (mm), 1 h maximum rainfall (mm); (4) Sunshine: day length (hr) and day length per year (MJ/m2); (5) Humidity: average humidity and minimum humidity; (6) Air pollution: SO_2_, CO, O_3_, NO_2_, and PM10. Statistics on meteorological factors are shown in Fig. 1B–G.

**Table 2:** Meteorological factors used in forecasting and their descriptions.

Group name	Feature name	Mean	Std.div	Min	Max
Temperature	Average temperature (°C)	13.1228	9.4744	− 6.2714	29.8286
	Average maximum temperature (°C)	18.5628	9.3420	− 2	35.5
	Maximum temperature (°C)	23.2809	8.5362	5.4286	39.4
	Average minimum temperature (°C)	8.3895	9.8835	− 11.3429	25.3429
	Minimum temperature (°C)	1.7086	10.8119	− 20.8714	20.5
Wind	Average wind speed (m/s)	1.9545	0.3890	1.1857	3.5286
	Maximum wind speed (m/s)	12.0837	2.4749	7.3857	20.5714
	Maximum instantaneous wind speed (m/s)	17.3248	3.0480	10.5286	28.3571
Rainfall	Average rainfall (mm)	3.5180	4.9080	0	32.4714
	Maximum rainfall (mm)	25.3245	29.2786	0	195.0143
	1 h maximum rainfall (mm)	8.8643	12.6071	0	59.6286
Day length	Day length (hr)	6.5254	1.8445	1.4571	12.0714
	Day length per year (MJ/m2)	13.7006	4.6451	4.5371	26.0343
Humidity	Average humidity (%)	67.5706	10.1568	44.2857	90.4286
	Minimum humidity (%)	22.8662	10.2869	6.2857	52.2857
Air pollution	SO_2_	0.0045	0.0012	0.0026	0.01042
	CO	0.4965	0.1158	0.3056	1.08039
	O_3_	0.0270	0.0091	0.0105	0.05755
	NO_2_	0.0224	0.0061	0.0085	0.03906
	PM10	44.9482	15.7274	16.2785	120.7358

### Data normalization

Normalization pre-processing is essential when there is a difference in the scale of features between the data. Min–max normalization converts all features between zero and one^22^. The min–max normalization equation is as follows:

$${x}{^\prime}=\frac{x-\mathrm{min}}{max-\mathrm{min}}$$

where $x$ denotes each feature value and $x{^\prime}$ denotes the change in scaling. Min and max are the maximum and minimum values of each feature.

### Estimation of suspected HFMD patients using DA-RNN

**Figure 5:**
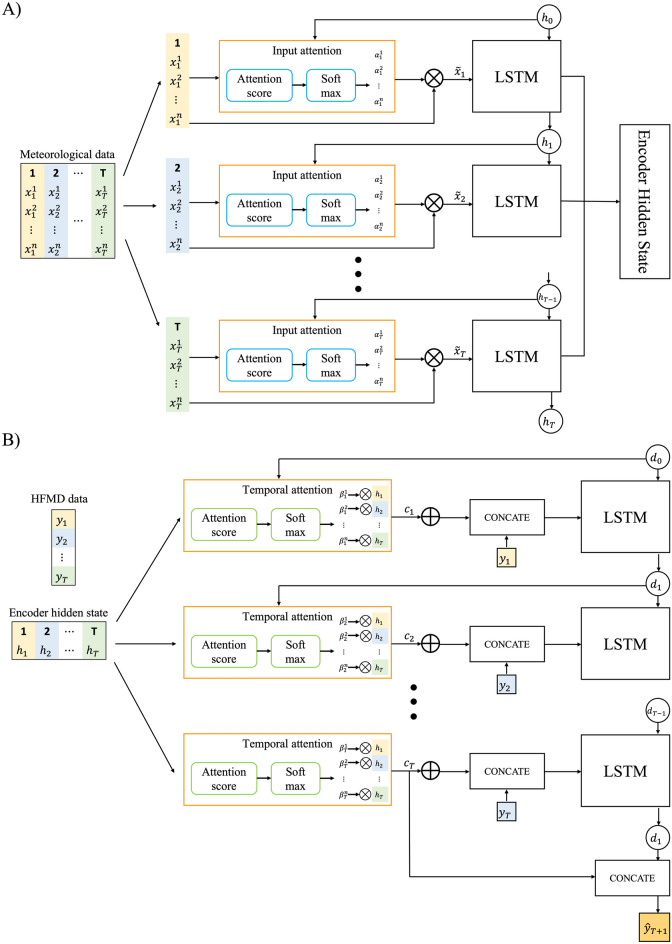
DA-RNN diagram. (**A**) Encoder (**B**) Decoder.

The traditional RNN successfully uses a time-series prediction algorithm, which has the problem of vanishing gradients. LSTM and GRU have been used to overcome this limitation. The LSTM and GRU are shown in Figure S3^23,24^. However, the same problem occurs when the time series increases. The structure of the encoder-decoder network was used; a representative example is Seq2Seq^25,26^. This structure has been extended to an attention mechanism that provides a score for past information because the performance degradation problem occurs when the input sequence is lengthy. Qin et al. proposed a DA-RNN as a time-series prediction model^18^. This structure overcomes the shortcomings of the RNN model in existing time-series studies using two attention mechanisms with encoder and decoder structures. The structure of this study’s model is shown in Fig. 5. The attention mechanism does not use the input data of each forecasting point in the same ratio but rather evaluates the attention score related to the data of the corresponding forecasting point and uses it for prediction. Among various attention scores, the Bahdanau (concate) method was used^26^.

This study compares the prediction results obtained using LSTM, GRU, and seq2seq to confirm the prediction performance of DA-RNN. The importance of the weather factor in the encoder and the temporal importance in the decoder were calculated. To confirm the influence of the importance calculation of each structure, the prediction results obtained through a single attention mechanism in the encoder and decoder were compared.

DA-RNN is an encoder-decoder-based algorithm comprising an attention mechanism in each encoder and decoder. The input data tensor consists of n driving series and n-1 target series during the T time step, and the output data are the T time step, which is called the target series of T time steps. Each encoder passes through the input attention and encoder LSTM structure. The input attention shown in Fig. 5 can be expressed mathematically as follows:

$${e}_{t}^{k}={v}_{e}^{T}\mathrm{tanh}\left({w}_{e}\left[{h}_{t-1};{s}_{t}\right]+{v}_{e}{x}^{k}\right),$$

$${\alpha }_{t}^{k}=\frac{\mathrm{exp}\left({e}_{t}^{k}\right)}{{\sum }_{i=1}^{n}\mathrm{exp}\left({e}_{t}^{i}\right)},$$

$$\widetilde{{x}_{t}}=\left({\alpha }_{t}^{1}{x}_{t}^{1},{\alpha }_{t}^{2}{x}_{t}^{2},\dots ,{\alpha }_{t}^{n}{x}_{t}^{n}\right),$$

where ${x}^{k}$ denotes the kth weather variable, ${h}_{t-1}$ denotes the hidden encoder state, and w denotes the encoder cell state. ${e}_{t}^{k}$ is calculated using the Bahdanau method as the kth attrition score of t time, and the attrition distribution ${\alpha }_{t}^{k}$ is calculated using softmax and calculated as the attention value $\widetilde{{x}_{t}}$ of t time. The mathematical expression of the encoder LSTM is expressed as follows:

$${f}_{t}=\sigma ({w}_{f}\left[{h}_{t-1};\widetilde{{x}_{t}}\right]+{b}_{f})$$

$${i}_{t}=\sigma \left({w}_{i}\left[{h}_{t-1};\widetilde{{x}_{t}}\right]+{b}_{i}\right)$$

$${o}_{t}=\sigma \left({w}_{o}\left[{h}_{t-1};\widetilde{{x}_{t}}\right]+{b}_{o}\right)$$

$$s_{t} = f_{t} \odot s_{t - 1 } i_{t} + {\text{tanh}} \odot \left( {w_{s} \left[ {h_{t - 1} ;\widetilde{{x_{t} }}} \right] + b_{s} } \right)$$

$$h_{t} = o_{t} \odot{\text{tanh}} \left( {s_{t} } \right),$$

where $b$ denotes the bias, and the encoder LSTM has the same structure as the general LSTM. ${f}_{t}$ is a forget gate, ${i}_{t}$ is an input gate, ${o}_{t}$ is an output gate, ${s}_{t}$ is a cell state, and ${h}_{t}$ is a hidden state. $\sigma$ and $\odot$ are sigmoid function and element wise multiplication, respectively.

The decoder has a temporal attention and an LSTM decoder structure. The temporal attention is expressed mathematically as follows:

$${l}_{t}^{k}={v}_{d}^{T}\mathrm{tanh}\left({w}_{d}\left[{d}_{t-1};{s}_{t-1}{^\prime}\right]+{v}_{d}{h}_{i}\right)$$

$${\beta }_{t}^{i}=\frac{\mathrm{exp}\left({l}_{t}^{i}\right)}{{\sum }_{i=1}^{T}\mathrm{exp}\left({l}_{t}^{i}\right)}$$

$${\mathrm{c}}_{\mathrm{t}}={\sum }_{i=1}^{T}{\beta }_{t}^{i}{h}_{i}$$

$${\widetilde{y}}_{t-1 }={\widehat{w}}^{T}\left[{y}_{t-1};{c}_{t-1}\right]+\widetilde{b}.$$

The hidden state calculated using the encoder was used as the input for temporal attention. Similarly, the attention score ${l}_{t}^{k}$ was calculated using the Bahdanau method, and the attention distribution ${\beta }_{t}^{i}$ was calculated using the activation function softmax to obtain the temporal attention value ${\mathrm{c}}_{\mathrm{t}}$ at time t. In the LSTM decoder, the calculated attention value, and previous target value ${y}_{t-1}$ were concatenated and used.

The mathematical expression of the decoder LSTM is identical to that of the encoder LSTM, as expressed in the following equation.

$${f{^\prime}}_{t}=\sigma ({w{^\prime}}_{f} \left[{d}_{t-1};{\widetilde{y}}_{t-1}\right]+{b{^\prime}}_{f} )$$

$${i{^\prime}}_{t}=\sigma \left({w{^\prime}}_{i}\left[{d}_{t-1};{\widetilde{y}}_{t-1}\right]+{b{^\prime}}_{o}\right)$$

$${o{^\prime}}_{t}=\sigma \left({w{^\prime}}_{o}\left[{d}_{t-1};{\widetilde{y}}_{t-1}\right]+{b{^\prime}}_{s}\right)$$

$$s_{t} ^{^\prime} = f_{t} ^{^\prime} \odot s_{t - 1{^\prime}} i{^\prime}_{t} + tanh \odot \left( {w{^\prime}_{s} \left[ {d_{t - 1} ;\tilde{y}_{t - 1} } \right] + b{^\prime}_{s}} \right)$$

$$d_{t} = o{^\prime}_{t} \,tanh \odot \left( {s{^\prime}_{t} } \right)$$

$$\widehat{{y}_{T}}={v}_{y}^{T}\left({w}_{y}\left[{d}_{T};{c}_{T}\right]+{b}_{w}\right)+{b}_{v}.$$

The DA-RNN model was configured using Python 3.9.7, PyTorch 1.11, Numpy 1.21.4, and Pandas 1.3.4.

### Accuracy analysis of prediction

Four evaluation scores—MAE, RMSE, MAPE, and R^2^—were computed to assess the effectiveness of the various methods for HFMD prediction. If ${y}_{T}$ is the actual value and $\widehat{{y}_{T}}$ is the value predicted by the methods, each score is mathematically expressed as follows:

$$MAE=\frac{1}{N}\sum_{i=1}^{N}|{y}_{T}^{i}-\widehat{{y}_{T}^{i} }|$$

$$RMSE= \sqrt{\frac{1}{N}\sum_{i=1}^{N}{\left({y}_{T}^{i}-\widehat{{y}_{T}^{i}}\right)}^{2}}$$

$$MAPE=\frac{1}{N}\sum_{i}^{N}|\frac{{y}_{T}^{i}-\widehat{{y}_{T}^{i}}}{{y}_{T}^{i}}|\times 100\%$$

$${R}^{2}= 1-\frac{\frac{1}{N}\sum_{i}^{N}{\left({y}_{T}^{i}-\widehat{{y}_{T}^{i}}\right)}^{2}}{\frac{1}{N}\sum_{i}^{N}{\left({y}_{T}^{i}-{\mu }_{y}\right)}^{2}}.$$

We set the accuracy scores metrics defined (MAE, RMSE, MAPE, R^2^).

### Sensitivity analysis

A drop group important measure was performed to determine the importance of the six meteorological groups. Accuracy scores metrics are calculated by removing each group of the meteorological group one by one. The smaller the calculated MAE, RMSE, and MAPE are, the better the performance is, but the closer R2 is to 1, the better the performance is. Therefore, the value of 1- R2 was used to provide the same standard.

To determine the order of important meteorological groups, we went through three steps. First, we calculated the important value to measure the accuracy scores for each meteorological group. Total group accuracy scores are values using all meteorological groups, and drop group accuracy scores are defined as accuracy scores with one group removed. The formula for calculating important value is as follows;

(Important value) = (total group accuracy scores) − (drop group accuracy scores).

After calculation, this important value was normalized to a minimum value of 0 and a maximum value of 1. The closer it is to 1, the higher the importance, and the closer it is to 0, the lower the importance.

Second, normalized important values are expressed as spider maps. We know that the group with the largest width in this spider map is the group with the highest importance. Thirdly, to determine the order of importance, we calculated the width of each group in the spider map to determine the order.

### Ethical considerations

This study analyzed publicly available HFMD and meteorological data^19–21^. Publicly available data with no personally identifiable information did not require ethical approval.

## Discussion

This study explores the meteorological factors that predict and influence the prevalence of HFMD in Korea. This study aimed to predict the number of suspected HFMD cases per 1,000 people per week and analyze the sensitivity of meteorological factors. We conducted HFMD prediction and sensitivity analysis using the time-series forecasting method DA-RNN. First, we estimated the number of suspected cases per 1000 people with HFMD from 2019 to 2020 using 2011 to 2018 data and all meteorological factors. To evaluate the performance of DA-RNN, we experimented with LSTM, GRU, Seq2seq, encoder attention-based seq2seq, and decoder attention-based seq2seq under the same experimental conditions. Second, MAE, RMSE, MAPE, and R^2^ were compared to determine the most influential group when meteorological factors were divided into groups based on temperature, wind, rainfall, sunshine, and humidity under the same conditions and when each group was excluded.

The DA-RNN outperformed other methods in terms of MAE, RMSE, MAPE, and R^2^ metrics (0.8544, 2.7117, 0.3163, and 0.9333, respectively). This was confirmed using single attention, demonstrating the importance of calculating the weights of the wake-up factors in the encoder attention mechanism. Using the sensitivity test results, among the six meteorological groups, rainfall, air pollution, wind and humidity groups were identified as groups of overall importance (see Supplementary Information).

According to GeoDetector theory, the meteorological factors affecting HFMD in Guangxi, an inland region of China, are similar to temperature and rainfall. However, because our country is surrounded by sea on three sides, not only rainfall but also humidity has a significant impact. This is consistent with a study in Japan, which has a similar topography, where relative humidity was found to be an important factor in HFMD^10^. We have added the day-length group and air pollution to examine the meteorological factors in previous studies. The prevalence pattern of HFMD from 2011 to 2019 and that in 2020 differed significantly, which indirectly confirms the great influence of the air pollution group on the degree of social activity of people owing to the impact of COVID-19.

This study has several limitations. First, the pattern of HFMD in 2020 changed significantly because of COVID-19. We chose the air pollution group as an indirect factor; however, the pattern was not sufficiently learned because of the lack of directly related data. Therefore, factors related to social phenomena and population density should be considered in future research. Additionally, the terrestrial viewpoint was not considered in this study. Therefore, it is necessary to establish a system that can evaluate risk levels by region and provide alerts. In this study, only meteorological factors were considered, which may have led to inaccurate forecasts, considering that other factors might be important. Subsequently, social and terrestrial factors should be considered to improve the accuracy of the DA-RNN model for HFMD prediction. To the best of our knowledge. This study is the first to use a DA-RNN for HFMD in Korea and reveals important meteorological factors. This model can significantly influence government policy by differentiating between the meteorological factors to be observed when predicting HFMD in Korea. This study’s framework could be extended to other epidemiological studies and time-series problems.

## Conclusion

This study proposes a new model to predict the number of suspected weekly HFMD cases using 20 meteorological factors. The meteorological factors were divided into six groups, and a sensitivity test was conducted to determine the most influential group. Our model uses the DA-RNN and shows good prediction results even in 2019 and 2020, which test period are difficult to predict compared with other models. The results showed that the factors that significantly affect HFMD are rainfall, air pollution, wind, humidity group. These results show the need for governments to consider meteorological factors in HFMD prevention guidelines.

### Supplementary Information

Supplementary Information.

## Data Availability

The meteorological dataset used in this study is available on the Korean Statistical Information Service website (https://kosis.kr/). The air pollution dataset used in this study is available on the Airkorea website (https://www.airkorea.or.kr/). The HFMD datasets used in this study are available on the Korea Centers for Disease Control and Prevention website (https://www.kdca.go.kr/npt/biz/npp/iss/hfmdStatisticsMain.do).
