# Supplementary material for: Dual-attention-based recurrent neural network for hand-foot-mouth disease prediction in Korea
Source: Sci Rep. 2023 Oct 3;13:16646. doi: 10.1038/s41598-023-43881-6 (PMC10547784; doi:10.1038/s41598-023-43881-6)
Supplement: Supplementary file 1 — Supplementary Information. [file 41598_2023_43881_MOESM1_ESM.docx]

| Group name | Feature name | Signification |
| --- | --- | --- |
| Temperature | Average temperature (℃) | Average of temperature. |
|  | Average maximum  temperature (℃) | Average of maximum temperature. |
|  | Maximum temperature (℃) | Maximum of temperature. |
|  | Average minimum  temperature (℃) | Average of minimum temperature. |
|  | Minimum temperature (℃) | Minimum of temperature for week. |
| Wind | Average wind speed (m/s) | Average of wind speed. |
|  | Maximum wind speed (m/s) | Maximum of wind speed. |
|  | Maximum instantaneous  wind speed (m/s) | Maximum instantaneous wind speed. |
| Rainfall | Average rainfall (mm) | Average of rainfall for week. |
|  | Maximum rainfall (mm) | Rainfall at the location with the most precipitation during the observed period. |
|  | 1hour maximum rainfall (mm) | Rainfall in places with the most precipitation during one hour of the observed day. |
| Day length | Day length (hr) | Cumulative time of sunlight per day. |
|  | Day length per year (MJ/m2) | The amount of radiant energy from the sun that reaches the ground. |
| Humidity | Average humidity (%) | Average relative humidity of 8 observations per day. |
|  | Minimum humidity (%) | Lowest relative humidity among 8 observations per day. |
| Air pollution | SO2 | Sulfurous acid gas generated from industrial processes |
|  | CO | The main source of carbon monoxide is the transportation sector. |
|  | O3 | Ozone is mainly emitted from automobiles and chemical processes. |
|  | NO2 | The main sources of nitrogen dioxide are automobiles and power plants. |
|  | PM10 | Dust with a diameter of 10𝜇𝑚 or less, which is emitted directly from industrial facilities, automobiles, heating and energy use, or generated by reaction in the atmosphere. |

Table S1. Meteorological characteristics and signification.


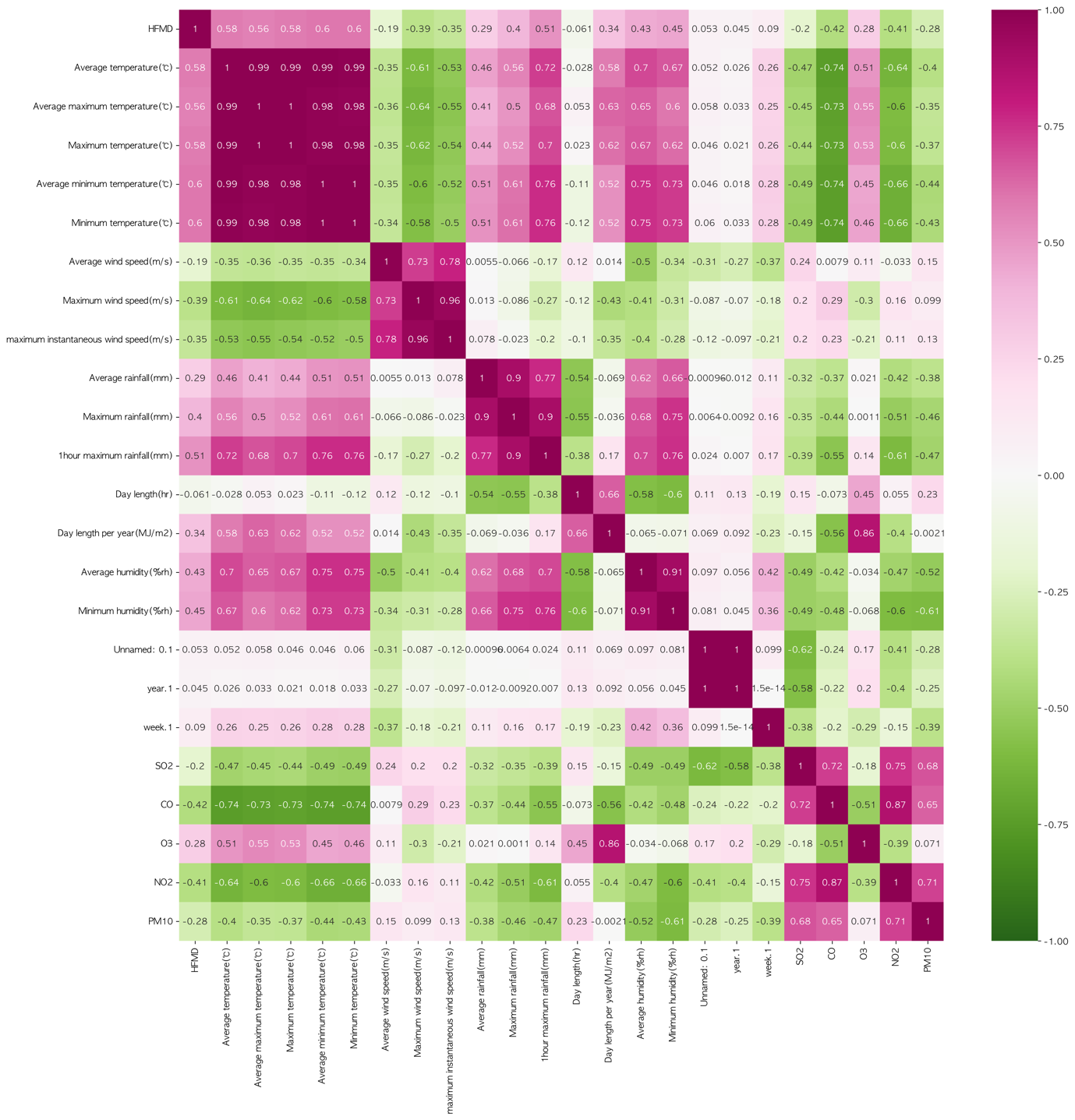


Figure S1. Correlation analysis of twenty meteorological factor with HFMD.

|  | MAE | RMSE | MAPE | R^2^ |
| --- | --- | --- | --- | --- |
| (-)Temperature | 1.3929 | 3.039 | 0.5131 | 0.9126 |
| (-)Wind | 1.2907 | 3.2931 | 0.3778 | 0.9016 |
| (-)Rainfall | 1.3084 | 3.2694 | 0.3955 | 0.903 |
| (-)Daylength | 1.1953 | 2.9387 | 0.3701 | 0.9216 |
| (-)Humidity | 1.3934 | 3.1278 | 0.4446 | 0.9112 |
| (-)AirPollution | 1.3205 | 3.2679 | 0.3647 | 0.9031 |
| total | 1.0811 | 2.9445 | 0.3686 | 0.9213 |

Table S2. Sensitivity test result of HFMD


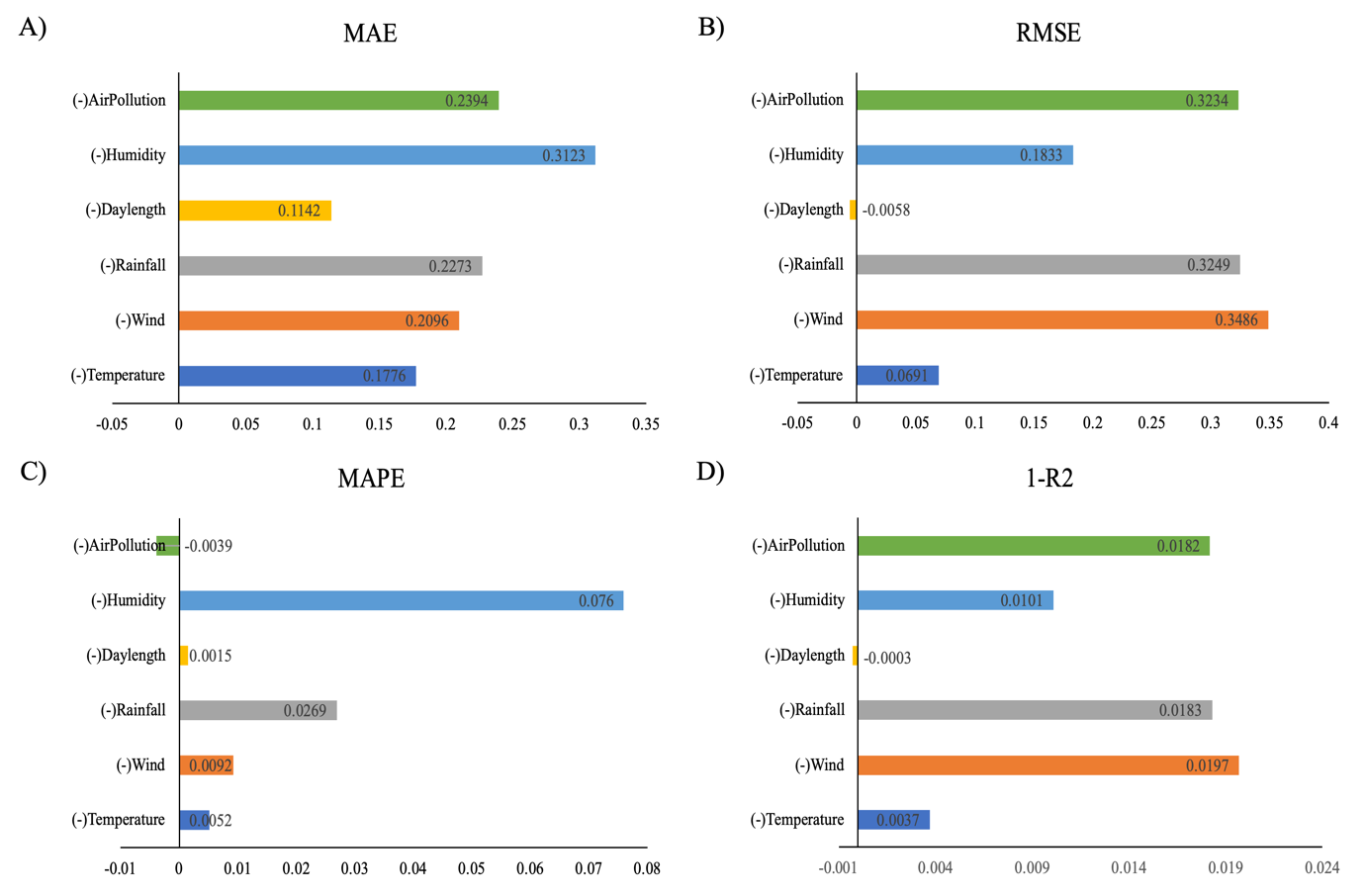


Figure S2. Sensitivity analysis important score results. (A) Important score of MAE; (B) Important score of RMSE; (C) Important score of MAPE; (D) Important score of 1- R^2^


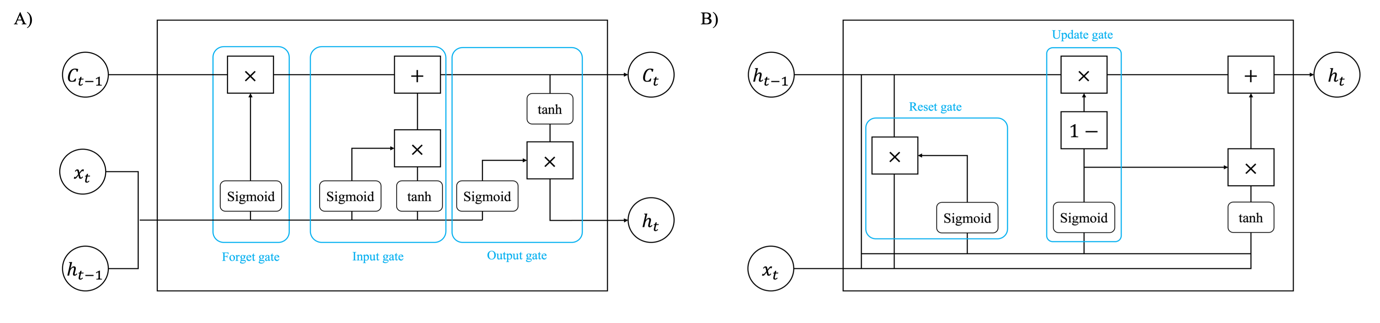


Figure S3. LSTM and GRU diagram. A) LSTM B) GRU.

LSTM

LSTM is a family of RNNs and was introduced by Hochreiter & Schmidhuber as machine learning [26]. This method is designed to solve the problem of vanishing gradients in long time series of RNNs. The difference from RNN is that it consists of three gates, input gate, forget gate, and output gate, to process long-term memory and short-term memory, rather than simply storing memory. LSTM is an iterative structure that stores overall past information using cell state, and hidden state is filtering the information needed at the current point into cell state. A picture of this structure is shown in Figure S3(A).

LSTM proceeds in the order of forget gate, input gate, and output gate. Forget gate is a gate for filtering past information. It is a structure in which sigmoid value ($f_{t}$) is obtained from current new information and past information, and if the value is 0, the past information is forgotten, and if it is 1, it is remembered. The input gate is a gate for filtering current information, and represents the degree and direction of memory selection for current information and past information with a sigmoid value ($I_{t}$) and a hyperbolic tangent value ($g_{t}$), respectively. The output gate calculates the final output value to be output.

The mathematical expression of LSTM is:

$$f_{t}=\sigma(w_{f} [h_{t-1};\tilde{x_{t}} ]+b_{f})$$

$$i_{t}=\sigma(w_{i} [h_{t-1};\tilde{x_{t}} ]+b_{i} )$$

$$o_{t}=\sigma(w_{o} [h_{t-1};\tilde{x_{t}} ]+b_{o} )$$

$$s_{t}=f_{t}⨀s_{t-1} i_{t}+tanh ⨀(w_{s} [h_{t-1};\tilde{x_{t}} ]+b_{s} )$$

$$h_{t}=o_{t}⨀tanh(s_{t})$$

b stands for bias. $f_{t}$ is a forget gate $i_{t}$ is an input gate $o_{t}$ is an output gate $s_{t}$ cell state $h_{t}$ is a hidden state.

GRU

GRU is a model that simply improves the structure of LSTM and was introduced by Junyoung Chung, Caglar Gulcehre, KyungHyun Cho and Yoshua Bengio[27]. The difference from LSTM is that three gates are composed of two gates, a reset gate and an update gate, and a hidden state is formed by combining a cell state and a hidden state. GRU also has a repetitive structure, and uses hidden states for memories in calculations at each gate. This structure is shown in Figure S3(B).

GRU proceeds in the order of reset gate and update gate. The reset gate is for filtering past information, and represents a value between 0 and 1 as the sigmoid value $\left( r_{t} \right)$ of the previous hidden state and the current information, and the value $\tilde{h_{t}}$ applied to the previous hidden state use. The update gate, similar to the input gate and forget gate of LSTM, filters past information and current information and calculates the reflection ratio for each. The current information ratio$\left( z_{t} \right)$is calculated as a sigmoid value of the previous hidden state and the current information, and the degree of subtracting this from 1 is selected as the past information reflection ratio. Outputs what is calculated at each gate as a hidden state, which is the current output value. The mathematical expression of GRU is as follows.

$$z_{t}=\sigma(W_{z} [h_{t-1};x_{t}]+b_{z} )$$

$$r_{t}=\sigma(W_{r} [h_{t-1};x_{t} ]+b_{r} )$$

$$\tilde{h_{t}} =tanh(W[r_{t}⨀h_{t-1};x_{t} ]+b_{h} )$$

$$h_{t}=(1-z_{t} )⨀h_{t-1}+z_{t}⨀\tilde{h_{t}}$$

$b$ stands for bias. $r_{t}$is the value filtered by the reset gate $\tilde{h_{t}}$, $z_{t}$ is the value obtained through the reset gate, $z_{t}$is the ratio of current information in the update gate, and $h_{t}$ is the hidden state.
